# Supplementary material for: Probing SARS-CoV-2 membrane binding peptide via single-molecule AFM-based force spectroscopy
Source: Nat Commun. 2025 Jan 2;16:6. doi: 10.1038/s41467-024-55358-9 (PMC11696146; doi:10.1038/s41467-024-55358-9)
Supplement: Supplementary file 1 — Supplementary Information [file 41467_2024_55358_MOESM1_ESM.pdf]

Supplementary Information

Probing SARS-CoV-2 Membrane Binding Peptide via Single-Molecule AFM-based Force Spectroscopy

Qingrong Zhang[1], Raissa S.L. Rosa[2], Ankita Ray[1], Kimberley Durlet[1], Gol Mohammad Dorrazehi[1], Rafael C. Bernardi[2,3,*] and David Alsteens[1,4,*]

[1]Louvain Institute of Biomolecular Science and Technology, Université catholique de Louvain, Croix du sud 4-5, L7.07.07, 1348 Louvain-la-Neuve, Belgium.
[2]Department of Chemistry and Biochemistry, Auburn University, 36849 Auburn, AL, U.S.A.
[3]Department of Physics, Auburn University, 36849 Auburn, AL, U.S.A.
[4]WELBIO department, WEL Research Institute, Avenue Pasteur, 6, 1300 Wavre, Belgium.

***Correspondence** should be addressed to R.C.B (rcbernardi@auburn.edu) and D.A. (david.alsteens@uclouvain.be)

This PDF contains Supplementary Figures 1 to 9

#1: BLI trace for kinetics estimation between MBP and DOPC/DPPC/Chol.

#2: Formation of lipid bilayer on mica solid support and estimation of height.

#3: Different tip chemistries for the preparation of functionalized AFM tips.

#4: Binding frequencies between AFM tip functionalized with different peptides and DOPC/Chol bilayer

#5: Local distance difference test scores and respective confidence predictions of MBP1 and MBP2.

#6: Average contact between residues and MBP2 as observed by simulations.

#7: Mass spectrometric analysis for MBP1 under oxidizing and reducing conditions.

#8: Analysis of lifetime and Bell's Fit.

#9: Extraction of lifetime for discrete force ranges.

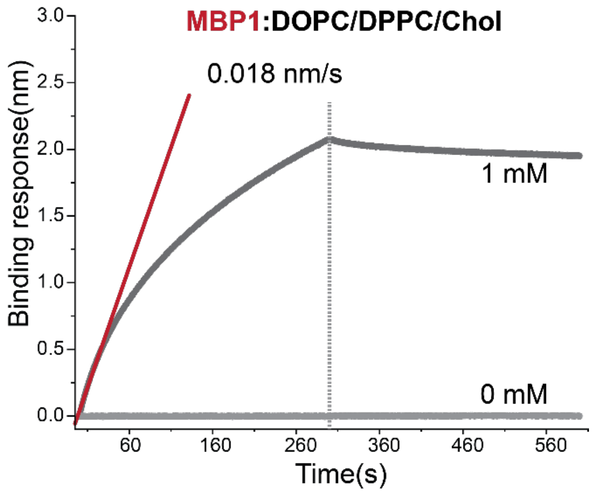

Supplementary Figure 1. BLI sensorgrams depicting the real-time binding of SARS-CoV-2 MBP and lipid vesicles consisting of DOPC/DPPC/Chol (40:40:20). Data are representative of three independent replicates.

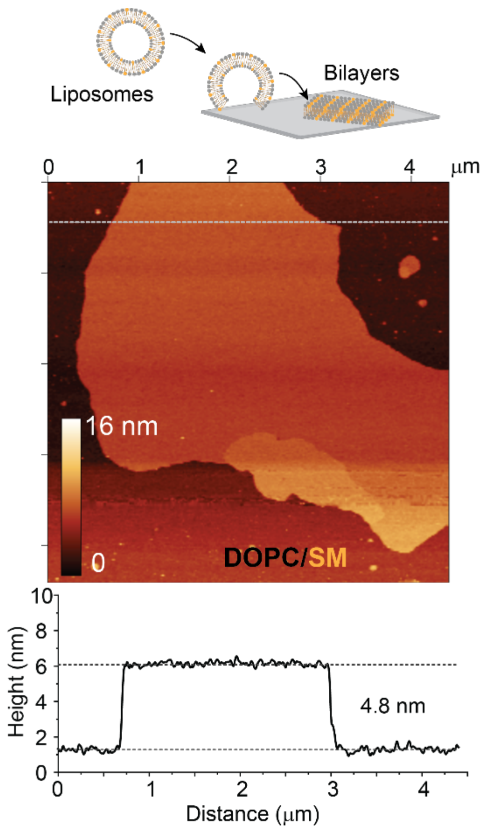

Supplementary Figure 2. Cartoon showing the formation of DOPC/SM bilayers bilayer on mica substrate (top). AFM height image of DOPC/SM bilayer (middle) and the corresponding height (bottom) was obtained by drawing a cross-section profile across the lipid bilayer. Data are representative of three independent replicates.

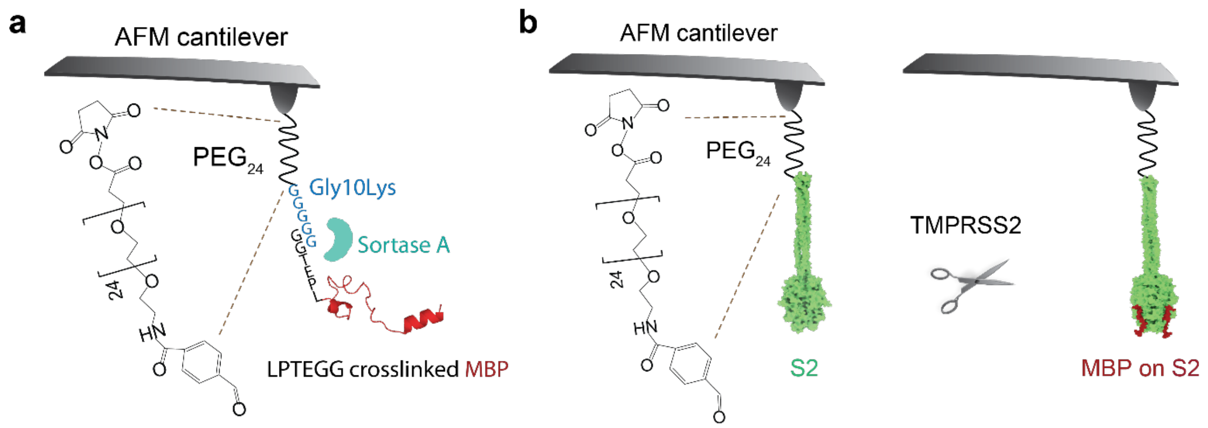

Supplementary Figure 3. Cartoon showing the different chemistries for the functionalization of AFM tips by MBP or S2-protein. The AFM tip is functionalized with a PEG spacer fused to either $Gly_{10}Lys$ peptides or S2 subunits, which in turn react with the LPETGG-MBP via the Sortase A enzyme **(a)** or treated by the TMPRSS2 **(b)**.

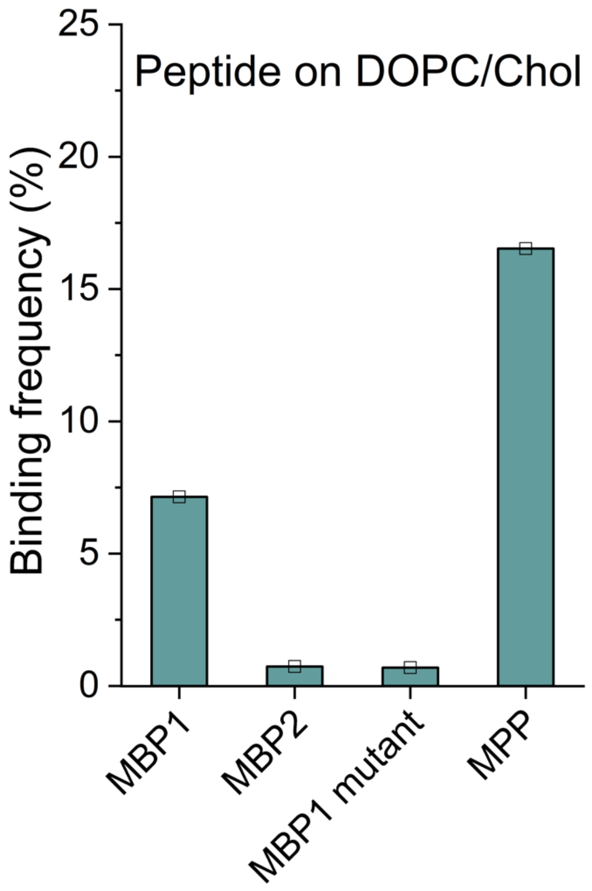

Supplementary Figure 4. Plot showing the quantification of specific binding events between different peptides tethered on the AFM tip and the DOPC/Chol bilayer. Data are representative of three independent replicates.

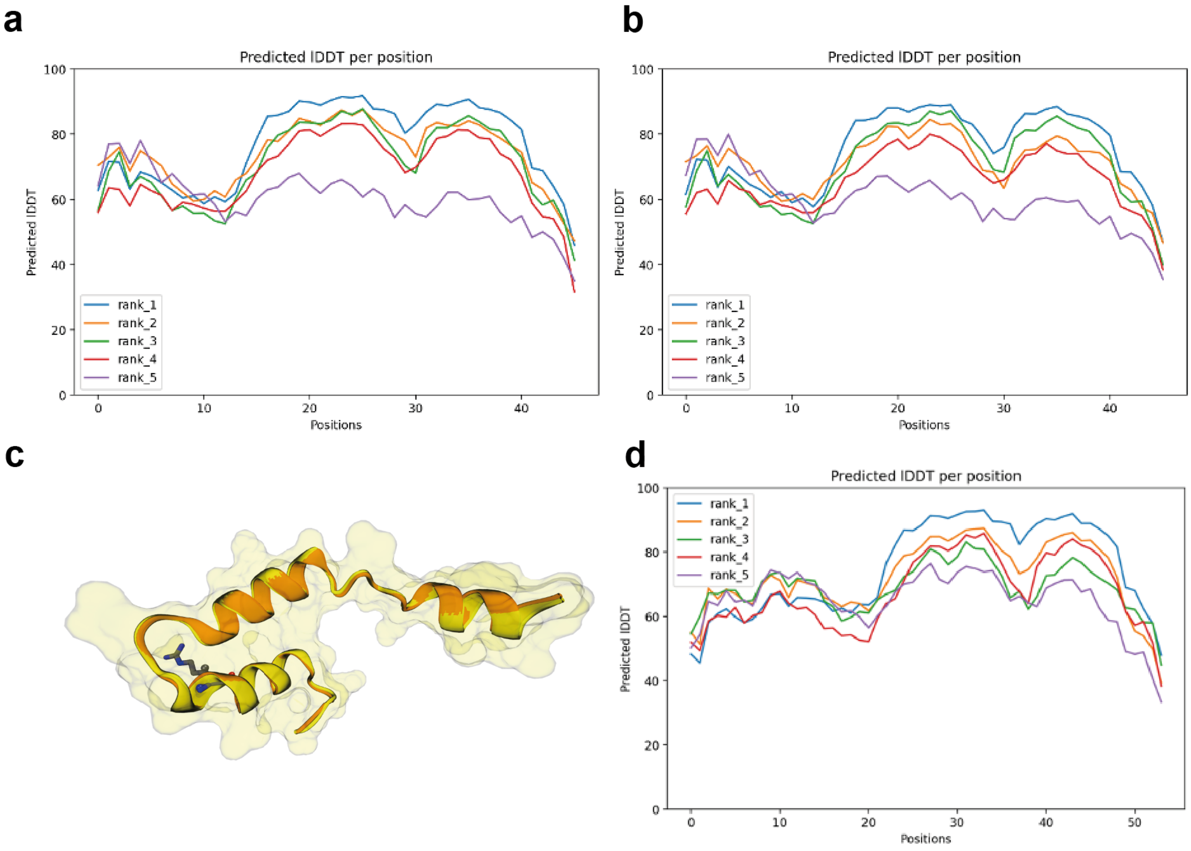

Supplementary Figure 5. Predicted IDDT scores for MBP1 (**a**), MBP1mutant (R846A) (**b**), and and MBP2 (**d**). The anticipated structures include atomic coordinates and confidence estimates for each residue, ranging from 0 to 100. Confidence increases with higher scores. The IDDT metric, a widely used tool in protein structure prediction, provides these confidence estimates. Residues with an IDDT score of ≥ 90 indicate exceptionally high confidence, while scores between 90 and 70 indicate high confidence. Scores between 70 and 50 suggest an OK structure that might need refinement, and scores below 50 correspond to very low confidence. As shown, the main helix regions exhibit very high confidence, whereas the linker regions are largely unstructured. All structures were refined using MD simulations. (**c**) 3D structure rendering of the wild-type peptide (yellow) and its R846A mutant (orange), calculated using AlphaFold2. For clarity, Arg846 in the wild-type is depicted as sticks, while Ala846 in the mutant is represented in ball-and-stick form. The root-mean-square deviation (RMSD) between the two peptide backbones is 0.386 $Å^2$.

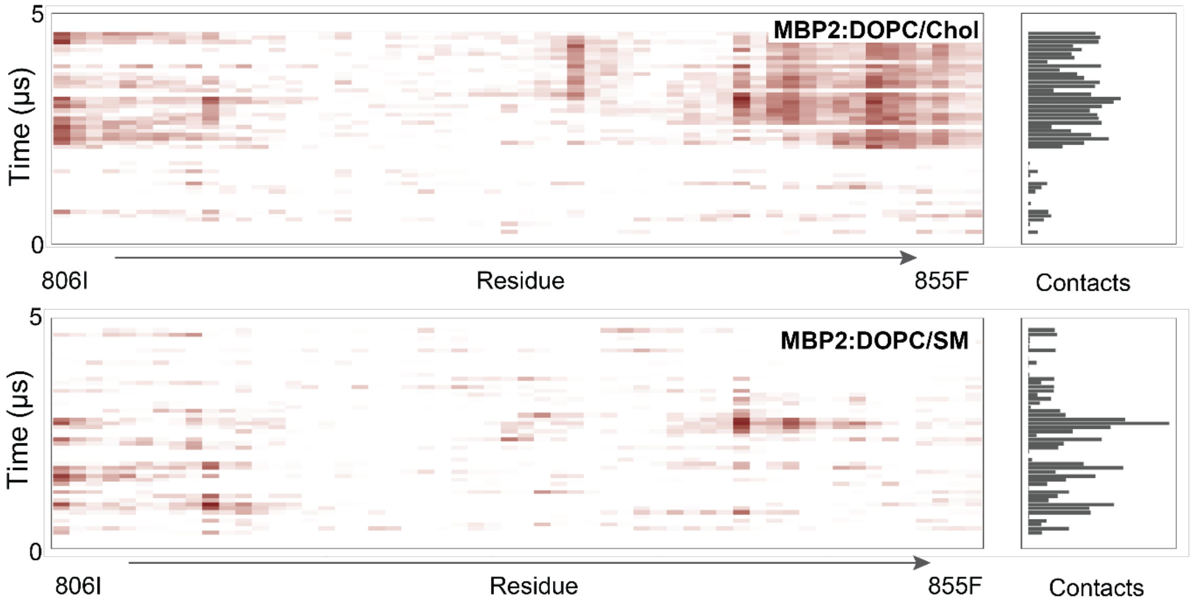

Supplementary Figure 6. Average contact between the amino acids of MBP2 and the membranes over time.

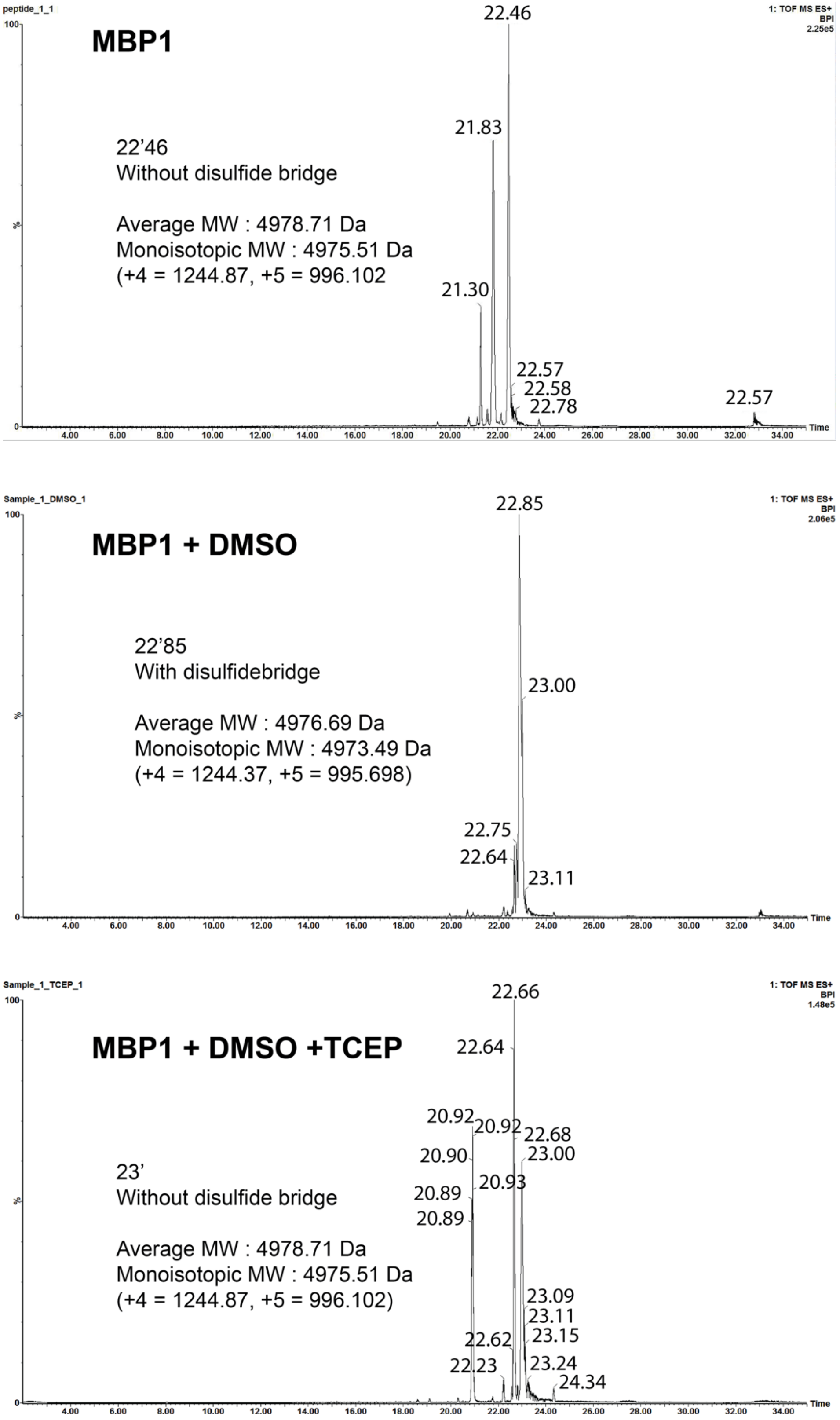

Supplementary Figure 7. Total ion chromatogram characterizing the formation (middle panel) and reduction (bottom panel) of the disulfide bridge by LC-MS in an ESI positive mode. The chromatogram was collected using a two-part linear gradient from 1% (v/v) ACN, 0.1% formic acid to 40% (v/v)) ACN, 0.1% formic acid for 15 min and from 40% (v/v) ACN, 0.1% formic acid to 85% (v/v)) ACN, 0.1% formic acid.

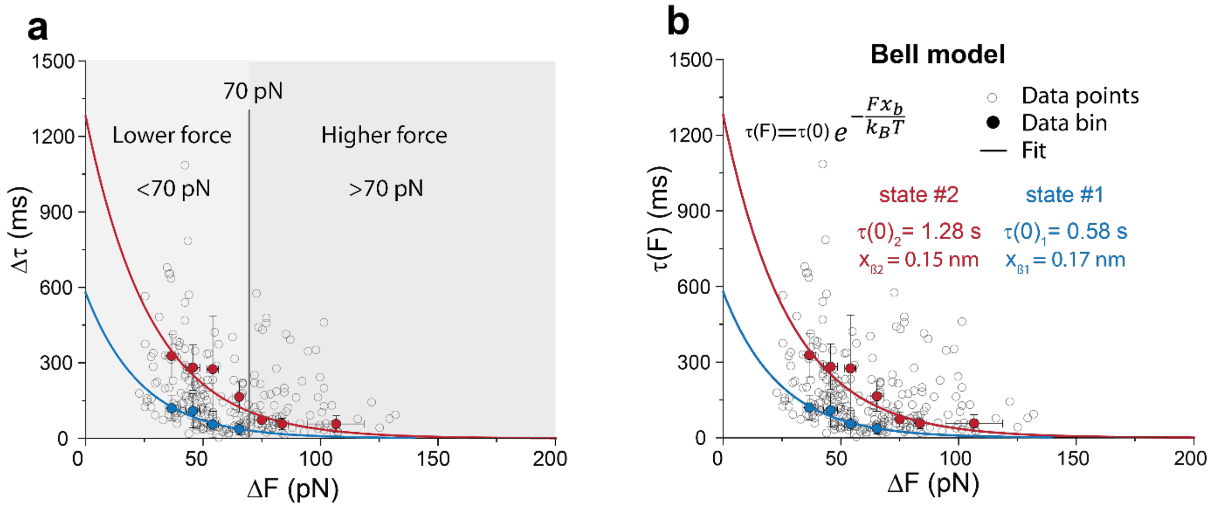

Supplementary Figure 8. (a) Distribution of lifetime obtained from the total force regime (range from 22-130 pN) reveals two distinct states at forces <70 pN and >70 pN. **(b)** Fitting lifetime against force of the two states performed either using the Bell model.

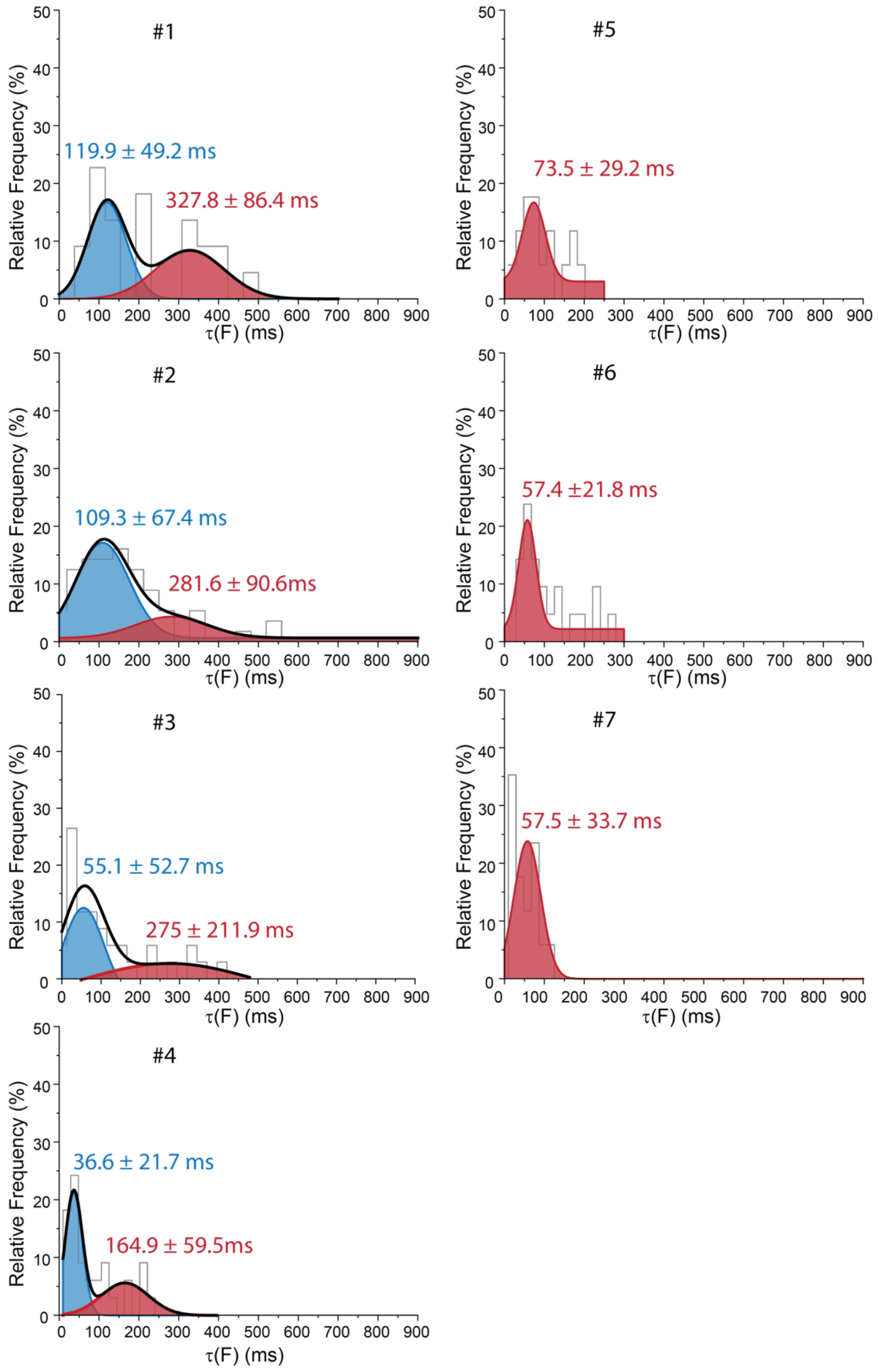

Supplementary Figure 9. Extraction of an average lifetime for discrete force regimes. Small force ranges #1–#7 are binned and the distributions of the lifetime are plotted as histograms. This classification reveals two peaks with average values corresponding to single (blue) or double (red) simultaneously established MBP-membrane interactions. This reveals two-lifetime peaks with average values corresponding to single (blue) and double (red) simultaneously established MBP-membrane interactions. The average values of the lifetime distributions are extracted and plotted on the lifetime against force, enabling their analysis using the Bell or DHS model (Fig. 5c, d or e).
