# Supplementary material for: Probing SARS-CoV-2 membrane binding peptide via single-molecule AFM-based force spectroscopy
Source: Nat Commun. 2025 Jan 2;16:6. doi: 10.1038/s41467-024-55358-9 (PMC11696146; doi:10.1038/s41467-024-55358-9)
Supplement: Supplementary file 2 — Description of Additional Supplementary Files [file 41467_2024_55358_MOESM2_ESM.pdf]

Description of Additional Supplementary Files

File Name: Supplementary Movie 1

Description: Movie illustrating the principle of AFM-based single-molecule force spectroscopy (SMFS) conducted in height-clamp mode
