# Supplementary material for: Probing SARS-CoV-2 membrane binding peptide via single-molecule AFM-based force spectroscopy
Source: Nat Commun. 2025 Jan 2;16:6. doi: 10.1038/s41467-024-55358-9 (PMC11696146; doi:10.1038/s41467-024-55358-9)
Supplement: Supplementary file 4 — Reporting Summary [file 41467_2024_55358_MOESM4_ESM.pdf]

nature portfolio

Corresponding author(s): Alsteens, David; Bernardi, Rafael

Last updated by author(s): Nov 5, 2024

Reporting Summary

Nature Portfolio wishes to improve the reproducibility of the work that we publish. This form provides structure for consistency and transparency in reporting. For further information on Nature Portfolio policies, see our Editorial Policies and the Editorial Policy Checklist.

Statistics

For all statistical analyses, confirm that the following items are present in the figure legend, table legend, main text, or Methods section.

n/a	Confirmed	
☐	☒	The exact sample size (*n*) for each experimental group/condition, given as a discrete number and unit of measurement
☐	☒	A statement on whether measurements were taken from distinct samples or whether the same sample was measured repeatedly
☐	☒	The statistical test(s) used AND whether they are one- or two-sided<br>*Only common tests should be described solely by name; describe more complex techniques in the Methods section.*
☒	☐	A description of all covariates tested
☒	☐	A description of any assumptions or corrections, such as tests of normality and adjustment for multiple comparisons
☐	☒	A full description of the statistical parameters including central tendency (e.g. means) or other basic estimates (e.g. regression coefficient) AND variation (e.g. standard deviation) or associated estimates of uncertainty (e.g. confidence intervals)
☒	☐	For null hypothesis testing, the test statistic (e.g. *F*, *t*, *r*) with confidence intervals, effect sizes, degrees of freedom and *P* value noted<br>*Give P values as exact values whenever suitable.*
☒	☐	For Bayesian analysis, information on the choice of priors and Markov chain Monte Carlo settings
☒	☐	For hierarchical and complex designs, identification of the appropriate level for tests and full reporting of outcomes
☒	☐	Estimates of effect sizes (e.g. Cohen's *d*, Pearson's *r*), indicating how they were calculated

Our web collection on statistics for biologists contains articles on many of the points above.

Software and code

Policy information about availability of computer code

Data collection	Nanoscope Software v9.1 (Bruker), QwikMD, molecular package GROMACS, VMD, AlphaFold2, NAMD 3, CHARMM-GUI, MassLynx V4.1
Data analysis	Nanoscope Analysis v1.7 (Bruker), Origin Software (OriginLab, version 2019), Octet Data Analysis 11.0, MassLynx V4.1, VMD, PyContact

For manuscripts utilizing custom algorithms or software that are central to the research but not yet described in published literature, software must be made available to editors and reviewers. We strongly encourage code deposition in a community repository (e.g. GitHub). See the Nature Portfolio guidelines for submitting code & software for further information.

Data

Policy information about availability of data

All manuscripts must include a data availability statement. This statement should provide the following information, where applicable:

- Accession codes, unique identifiers, or web links for publicly available datasets
- A description of any restrictions on data availability
- For clinical datasets or third party data, please ensure that the statement adheres to our policy

The Source data underlying Figs 1e, d, g h; 2c, d; 3b, c; 4d; 5d; 6b are provided as a source data file. All other relevant data are available from the corresponding authors upon reasonable request.

Research involving human participants, their data, or biological material

Policy information about studies with human participants or human data. See also policy information about sex, gender (identity/presentation), and sexual orientation and race, ethnicity and racism.

Reporting on sex and gender	N/A
Reporting on race, ethnicity, or other socially relevant groupings	N/A
Population characteristics	N/A
Recruitment	N/A
Ethics oversight	N/A

Note that full information on the approval of the study protocol must also be provided in the manuscript.

Field-specific reporting

Please select the one below that is the best fit for your research. If you are not sure, read the appropriate sections before making your selection.

☒ Life sciences ☐ Behavioural & social sciences ☐ Ecological, evolutionary & environmental sciences

For a reference copy of the document with all sections, see nature.com/documents/nr-reporting-summary-flat.pdf

Life sciences study design

All studies must disclose on these points even when the disclosure is negative.

Sample size	Typically, more than fifty data points are needed for single-molecule experiments. Here we are always above this value. Multiple replicates (as stated in the legends of the figures) were tested. Sample size was chosen based on data consistency.
Data exclusions	No data was excluded from the analysis.
Replication	The experiments were sucessfully replicated at least 3-5 times.
Randomization	Not applicable
Blinding	Not applicable

Reporting for specific materials, systems and methods

We require information from authors about some types of materials, experimental systems and methods used in many studies. Here, indicate whether each material, system or method listed is relevant to your study. If you are not sure if a list item applies to your research, read the appropriate section before selecting a response.

Materials & experimental systems

n/a	Involved in the study	
☐	☒	Antibodies
☐	☒	Eukaryotic cell lines
☒	☐	Palaeontology and archaeology
☒	☐	Animals and other organisms
☒	☐	Clinical data
☒	☐	Dual use research of concern
☒	☐	Plants

Methods

n/a	Involved in the study	
☒	☐	ChIP-seq
☒	☐	Flow cytometry
☒	☐	MRI-based neuroimaging

Antibodies

Antibodies used	Anti-6X His tag® antibody (Rabbit, iGG, polyclonal, Abcam ab9108)
Validation	Abcam ab9108 validation was performed by the manufacturer: ELISA: Antibody specificity was verified by ELISA against peptide

Validation	conjugated to BSA (HHHHHH-BSA). A 1:25,000 dilution of the antibody gave an OD=1.0 in a 15 minute reaction using HRP-conjugated Goat anti-Rabbit IgG at 1:20,000 and TMB as the substrate. Appropriate specificity controls were run.

Eukaryotic cell lines

Policy information about cell lines and Sex and Gender in Research

Cell line source(s)	HEK293T (CRL-3216), A549 (CCL-185) used to express ACE2 and TMPRSS2
Authentication	The cells obtained from ATCC were authenticated prior to purchase using STR profiling. The replicates of each experiment were conducted with cells from different passages, and no significant difference was observed among these replicates.
Mycoplasma contamination	We did not see any indicators of mycoplasma contamination and the test showed no contamination.
Commonly misidentified lines (See ICLAC register)	The cell lines used are not listed as commonly misidentified in the ICLAC register.

Plants

Seed stocks	N/A
Novel plant genotypes	N/A
Authentication	N/A
